# The relationship between social media usage and loneliness among younger and older adults: the moderating effect of shyness

**DOI:** 10.1186/s40359-024-01727-4

**Published:** 2024-06-11

**Authors:** Ya-Ling Wang, Yi-Jia Chen, Chih-Chi Liu

**Affiliations:** Department of Adult and Continuing Education, National Taiwan Normal University, Taipei, Taiwan

**Keywords:** Social media usage, Older adults, Shyness, Loneliness

## Abstract

Does social media alleviate or exacerbate loneliness? Past research has shown mixed results regarding the relationship between social media usage and loneliness among younger and older adults. Unlike younger individuals, older adults may decrease their loneliness through social media interactions. Additionally, previous research has indicated that the link between social media use and loneliness can vary depending on one’s shy tendency. Therefore, this study aims to explore the relationship between individuals’ social media use and loneliness while considering age and shyness tendency as moderating variables. The study employed a questionnaire survey conducted through convenience sampling, resulting in 234 valid responses from participants in Northern Taiwan. Among them, 113 were college students (aged 18 to 25, average age 19.40), and 121 were older adults (aged 50 to 82, average age 60.81). Using hierarchical regression analysis, results indicated that (1) age moderates the relationship between personal social media use and loneliness. Minimal differences were observed among younger individuals, but among older adults, increased social media usage time was associated with a significant reduction in loneliness. (2) Shyness tendency moderate the relationship between personal social media use and loneliness. Individuals with higher shyness tendency experience an increase in loneliness as their social media usage time lengthens.

## Introduction

With the advancement of technology, interpersonal interactions have evolved beyond physical social spaces, extending into the realm of virtual social networks. According to a survey by InsightXplorer [1], Taiwan ranks third in internet usage among Asian countries, following only Japan and South Korea. It is estimated that there are approximately 18.66 million internet users in the country, with an overall internet penetration rate of 79.2%. Moreover, even among individuals aged 55 to 64, over 60% are internet users. In the same year, Taiwan officially transitioned from an aging society to an aged society, making the use of information technology among older adults an increasingly important topic of study.

As the number of users and time spent on social network sites (SNS) continues to grow, research in this area has expanded. Various studies have focused on different social media platforms. When taking into account geographical preferences, Twitter seems to be more popular among Americans. However, recent studies have noted a rising interest in Instagram among different generations (e.g., [2–4]).

Among older adults, Facebook is the preferred and the most commonly used social media platform [5]. However, even though Instagram is primarily used by young people, Facebook remains an important social media platform in studies of the social behavior of the younger generation. For example, research on the Hong Kong civil movement by Agur and Frisch [6] and on the Taiwanese student movement by Tsatsou [7] both centered on Facebook as the primary social media platform. Therefore, for a comprehensive comparison of social media usage between Taiwanese young people and older adults, Facebook is considered as a suitable choice, especially given its current ranking as the most widely used social media platform in Taiwan [1].

In studies related to Facebook usage, Kross et al. [8] conducted an experience-sampling study in which they asked university students about their frequency of current Facebook use over a two-week period. The study found that participants’ life satisfaction gradually decreased during the two weeks of Facebook use. University students often use social networking sites to stay in touch with friends, and excessive time spent online or on social media can lead to increased feelings of loneliness [9]. However, for older adults, using social networking sites may have potential benefits. Research indicates that older adults are more likely to experience social isolation issues, such as reduced communication with colleagues after retirement, leading to feelings of loneliness [10, 11]. Nevertheless, the internet can facilitate communication and interaction with others among older adults. As Jung et al. [12] pointed out, older adults use Facebook for various reasons, including connecting with people they wouldn’t usually have contact with, sharing photos, passively staying in touch with friends and family, and participating in convenient organizational and communication groups. However, previous research has primarily focused on specific age groups, with fewer studies simultaneously comparing the psychological well-being of Facebook users across different age groups. Therefore, this study aims to contribute to this gap in the literature.

In addition to age, an individual’s experience of loneliness when using social networking sites may also be influenced by differences in shyness tendency. Shyness tendency refer to an individual’s feelings of nervousness, anxiety, or other awkward discomfort when interacting with others [13, 14]. People who are easily shy may face obstacles in interpersonal relationships and have difficulty integrating into social situations [14]. In the context of the internet era, individuals with shyness tendency may also face obstacles in online social interactions, leading to feelings of loneliness. Research by Frison and Eggermont [15] found that individuals who can establish stable relationships online are more likely to reduce negative feelings. Additionally, the study by Sheldon [16] showed that users of Facebook with shyness tendency experience lower levels of loneliness. Furthermore, extroverted individuals tend to use social media more frequently [17]. Therefore, shyness tendency may be an important influencing factor in social networking site usage.

In summary, the association between social networking site usage and loneliness varies not only by age but also by individual shyness tendency. Thus, this study aims to explore the relationship between Facebook usage and loneliness in-depth, using age and shyness tendency as moderating factors. Accordingly, the objectives of this study are as follows: (1) to investigate whether age moderates the relationship between Facebook usage time and loneliness, and (2) to explore whether shyness tendency moderate the relationship between Facebook usage time and loneliness.

## Literature review

### Definition and related research on loneliness

This study defines loneliness based on the synthesis by Peplau and Perlman [18] as a negative experience that elicits aversion and unpleasantness (e.g., hostility towards others) [19–21] and the inability to satisfy one’s need for intimacy in relationships (e.g., family, friendship) [22]. Past research has proposed various explanations for the causes of loneliness. The first significant factor is the lack of companionship from friendships [23]. During childhood, forming friendships and the quality of those friendships are crucial for preventing loneliness. Lack of companionship from friends during this period can lead to increased feelings of loneliness. As individuals age, the absence of a sense of belonging to a social group can also contribute to increased loneliness [24, 25]. Lack of friendship, low-quality friendships, or rejection and bullying by peer groups are all factors that contribute to loneliness during adolescence [11].

The second factor contributing to loneliness is the lack of or dissatisfaction with romantic relationships. During adolescence and young adulthood (e.g., college years), in addition to the importance of friendship support, individuals begin to place increasing emphasis on romantic relationships [26, 27]. Previous studies involving college students have found a correlation between high satisfaction with romantic relationships and reduced loneliness, while disappointment in romantic relationships leads to increased loneliness [28]. Furthermore, marital status in later life can also predict feelings of loneliness [29].

Additionally, besides the aforementioned factors related to friendship and romantic relationships causing increased loneliness, research on the relationship between older adults and loneliness has identified factors such as physical and mental health decline, the loss of a partner, and increasing social disconnection as contributors to elevated loneliness. Dykstra et al. [30] studied individuals aged 55 and above and found that as age increases, feelings of loneliness also rise. Loneliness can be exacerbated by the loss of a partner or declining physical health. Courtin and Knapp [31], in a literature review on social isolation and loneliness among older adults and their impact on physical and mental health found that older adults experiencing social isolation and loneliness are at risk for depression and cardiovascular health issues. Theeke [32] studied the relationship between health and loneliness risk in people aged 50 and above, revealing that individuals who experience prolonged loneliness engage in less physical activity, have more chronic health problems, and are more likely to experience depression. Victor and Bowling [33] conducted a longitudinal study on older adults and found that loneliness not only affects physical and mental health but is also related to changes in marital status, lifestyle arrangements, and personal social network patterns.

### Measurement of loneliness

Scholars have proposed various measurement methods for loneliness. For example, Russell [34] defined loneliness as a unidimensional concept and developed the UCLA Loneliness Scale Version 3 (UCLA-3) using a 4-point Likert scale for measurement. However, since this study aims to measure loneliness related to interactions with different individuals, the UCLA-3 scale was not used in this study. Weiss [22] was the first to differentiate loneliness into multiple dimensions. Loneliness was divided into social loneliness and emotional loneliness. Social loneliness refers to an individual’s inability to establish good relationships with others, resulting in feelings of isolation. Emotional loneliness refers to a lack of intimate relationships (e.g., a partner) and a lack of emotional connection or dependence on others. Of the two, emotional loneliness, where emotional needs are unmet, tends to result in greater loneliness.

DiTommaso and Spinner [35] not only validated Weiss’s [22] concept but also further divided emotional loneliness into romantic loneliness and family loneliness. They developed the Social and Emotional Loneliness Scale for Adults (SELSA), which consists of a total of 37 items. In 1997, a short version of the SELSA was developed from the original scale, known as the Short Form of the Social and Emotional Loneliness Scale for Adults (SELSA-S) [35]. Other researchers have verified the stability of this scale with different populations (college students, military personnel, and individuals with mental illnesses), with Cronbach’s alpha ranging from 0.87 to 0.90 [36]. Letts [37] also used this scale and found good reliability in a study with older adults (ages 55–88). Given the stability of the SELSA-S scale in previous studies with both college students and older adults, this study adapted the SELSA-S and made modifications to create a scale suitable for its research purposes.

In summary, the causes of loneliness may change with age, and the primary sources of loneliness may differ among different age groups. However, the main causes often relate to dissatisfaction in friendships, romantic relationships, and family relationships. Since this study aims to measure loneliness in both college students and older adults, the sources of loneliness were combined for measurement during data collection to account for the potential direct influence of age on loneliness.

### Research on social media use and loneliness

In modern society, people often face psychological issues related to loneliness. Previous research has shown that adults sometimes experience loneliness, with 6% of the population believing that they feel lonely all the time [38]. Loneliness appears to be on the rise in today’s society [33, 38]. However, with the advent of the internet, virtual spaces have become available for people to interact, leading to numerous studies exploring the relationship between social media use and loneliness. Research indicates that using social media for communication and interaction with others can reduce feelings of depression and loneliness. In empirical studies, Kross et al. [8] examined Facebook usage and found that interactions on Facebook, such as messaging, posting, and receiving responses, were associated with decreased depressive emotions. Additionally, posting new status updates on Facebook, regardless of receiving replies, was linked to reduced loneliness within a week [39].

Furthermore, Burke and Kraut [40] conducted a month-long longitudinal study involving 1,910 Facebook users and questions about their subjective well-being. They found that prolonged conversations with close friends on Facebook were associated with increased feelings of happiness. Burke [41] also noted that engaging in communication with others on public platforms within social media reduced feelings of loneliness. In other words, using social media for communication and chatting with others could enhance subjective well-being and reduce feelings of loneliness. Conversely, passive information consumption (e.g., browsing, shopping) on social media could lead to negative psychological responses, such as depression and loneliness. From empirical research, Verduyn et al. [42] found that passive Facebook use could trigger jealousy and decrease happiness. Tandoc et al. [43] also pointed out that browsing Facebook and experiencing jealousy could increase depressive and negative emotions. Additionally, Guo et al. [44] discovered that using the entertainment features of social media could increase an individual’s feelings of loneliness.

### Generational difference in social media use and loneliness

The relationship between loneliness and internet use varied across different age groups [45]. Research has shown diverging patterns in late adolescence and adulthood, but in studies involving older adults, social media use has been found to reduce feelings of loneliness. Therefore, the following will separately examine research on young adults and older adults regarding their use of social media and its association with loneliness, leading to hypothesis inferences.

Previous research has indicated that young adults are more active on social media platforms [46]. Spending more time on social media has been associated with negative emotions such as depression, loneliness, and lower life satisfaction [8, 9, 47]. This might be because college students are prone to engage in social comparison on social media platforms [46]. Social comparison theory suggests that individuals, in the absence of objective information, use others as a yardstick for self-evaluation [48]. In recent years, with the rise of social media, the concept of “Facebook depression” has been proposed, implying that excessive engagement with social media can have negative effects, especially among young people [49]. Relevant studies have found that investing more effort and time into social networking sites is associated with higher levels of depressive emotions [50]. Kross et al. [8] conducted an experience-sampling study in which they inquired about the frequency and feelings of college students’ Facebook use over a two-week period. They found that participants experienced a gradual decrease in life satisfaction during this time. With the advancement of technology, smartphones have become a common means of accessing social media content and messages. Lemieux et al. [47] investigated Facebook use among college students and found that spending more time on Facebook was associated with increased feelings of loneliness. Peper and Harvey [51] studied smartphone addiction among college students and found that higher usage frequency was linked to higher levels of negative emotions such as loneliness, anxiety, and depression. Chen [52] proposed that college students who use the internet more frequently, spend longer periods online, and have higher expectations for online opposite-sex friendships tend to experience higher levels of real-life loneliness. Based on the above findings, it can be inferred that young people who invest more effort, time, and frequency into social media tend to experience higher negative emotions, such as depression, loneliness, and lower life satisfaction.

As technology has evolved, the number of older adults using the internet has been steadily increasing. During this stage of life, older adults often experience a reduced social circle due to retirement. On social media, unlike young people who engage in social comparison, older adults typically focus on family-related matters or one-on-one interactions [46, 53]. Most studies indicate that prolonged use of technology products and the internet can reduce feelings of loneliness among older adults [54]. This is because interaction with others through technology can enhance social support for older adults and improve their cognitive functions [55]. Choi et al. [56] proposed that using technology products can enhance social support among older adults through activities such as video calls with family or friends [57], communication [58, 59], or simply learning how to use technology products [60–62]. In summary, age differences may lead to variations in the degree of loneliness, with young people experiencing increased loneliness with social media use and older adults experiencing decreased loneliness. Therefore, the following hypotheses are proposed:

#### H1

Age differences will moderate the relationship between individual social media use and loneliness.

#### H1-1

Younger individuals who spend more time on Facebook will experience increased loneliness.

#### H1-2

Older individuals who spend more time on Facebook will experience decreased loneliness.

### Effects of shyness on the relationship between social media use and loneliness

The causes of loneliness can be attributed not only to the dissatisfaction individuals may feel in their real versus expected social relationships, and the quantity and quality of their social interactions but also to differences in personality traits [18]. This study aims to explore the relationship between shyness and loneliness, and the following will mainly elaborate on the relevant content.

Zimbardo et al. [63] pointed out a significant relationship between shyness and loneliness. Individuals with higher shyness tendency tend to have higher self-consciousness [64, 65], which means that they are more concerned about how others perceive them, leading to self-protective behaviors [66], lower self-esteem [13, 64], and emotional issues such as anxiety and depression [63]. People with higher levels of shyness tend to experience negative impacts on their lives, including lower subjective well-being, life satisfaction, and overall quality of life [67–69].

According to past research, Bian and Leung [70] studied smartphone addiction and usage patterns, which indicated that individuals who spent extended periods on their smartphones browsing social media, and sending and receiving messages, were more prone to shyness and experienced higher levels of loneliness. Satici [71] also found that individuals addicted to Facebook, as shyness and loneliness levels increased, reported decreased subjective well-being. In other words, individuals with higher shyness tendency experienced increased feelings of loneliness as they spent more time on Facebook.

In contrast, for individuals with lower shyness tendency who use Facebook, previous research suggests that they tend to have more extroverted personalities and lower levels of narcissism, leading to lower feelings of loneliness [72]. Zhou et al. [73] studied the online behavior of introverted and extroverted individuals and found that extroverted individuals were more likely to express both positive and negative emotions online. In contrast, introverted individuals posted more negative emotion-related content, expressing anger, fear, and disgust. In other words, individuals with lower shyness tendency are more capable of expressing their positive or negative emotions as needed, thereby reducing feelings of loneliness. In summary, this study posits that as users spend more time on Facebook, those with higher shyness tendency will experience increased loneliness, whereas those with lower shyness tendency will experience decreased loneliness. Based on the aforementioned theories and research, this study proposes the following hypotheses:

#### H2

Shyness tendency will moderate the relationship between individual social media use and loneliness.

#### H2-1

Individuals with higher shyness tendency who spend more time on Facebook will experience increased loneliness.

#### H2-2

Individuals with lower shyness tendency who spend more time on Facebook will experience decreased loneliness.

## Method

### Participants

This study employed convenience sampling. The pilot and formal questionnaires for young adults were distributed in a classroom at a university in the northern region. However, the pilot and formal questionnaires were distributed in different courses with non-overlapping student lists. For older adults, the pilot and formal questionnaires were distributed at a community college in the northern region, with no duplication in the completion of the pilot and formal questionnaires.

The pilot questionnaires serve the purpose of ensuring the reliability and validity of the questionnaire content. Additionally, it aids in compiling the formal questionnaire by analyzing the results obtained from the pilot questionnaire. A total of 70 valid pilot questionnaires were collected from college students, aged between 19 and 24 years. Among them, there were 20 males (28.6%) and 50 females (71.4%), with an average age of 20.70 years and a standard deviation of 1.20. For older adults, 22 valid pilot questionnaires were collected, with ages ranging from 48 to 80 years. Among them, there were 2 males (9.1%) and 20 females (90.9%), with an average age of 64.05 years and a standard deviation of 6.74. In total, 92 valid pilot questionnaires were collected, including 22 males (23.9%) and 70 females (76.1%).

A total of 113 valid formal questionnaires were collected from university students in the northern region, ranging in age from 18 to 25 years. Among them, there were 26 males (23%) and 87 females (77%), with an average age of 19.40 years and a standard deviation of 1.33. For older adults, 121 valid formal questionnaires were collected, with ages ranging from 50 to 82 years. Among them, there were 38 males (31.4%) and 83 females (68.8%), with an average age of 60.81 years and a standard deviation of 5.80. In total, 234 valid formal questionnaires were collected, including 64 males (27.4%) and 170 females (72.6%).

### Measures

In this study, participants were asked to self-report their social media usage. Specifically, they were asked about the total time (in minutes) spent on Facebook in a day. A longer duration indicates that individuals spend more time on social media. Example question: " Could you please take a moment to reflect and share with us the average amount of time you spend on Facebook per day?”

Additionally, this study assessed the level of loneliness using a modified version of the Social and Emotional Loneliness Scale for Adults - Short Form (SELSA-S), based on DiTommaso et al. [36].Based on the current research objectives, this study opted to include the Social and Family subscales from the SELSA-S while excluding the Romantic subscale, as it is less pertinent to the study’s scope of focus. The scale used a 5-point Likert scale to measure the degree of loneliness. Each question was rated on a scale from “strongly disagree” (1) to “strongly agree” (5), with scores ranging from 1 to 5. There was a total of 9 questions, including reverse-scored items. Higher scores indicated a higher level of perceived loneliness. Sample items included “I don’t have any friends who share my views, but I wish I did” and " I feel alone when I am with my family.”

Finally, this study aimed to investigate both adult college students and older adults. For this purpose, a modified version of the Shyness and Social Orientation Scale for Adults, based on Asendorpf & Wilpers [74], was used to assess shyness tendency. According to the literature review conducted for our study, we are specifically examining the moderation effect of Shyness Tendency. Consequently, we have made adjustments to the Shyness and Social Orientation in Adults scale by Asendorpf and Wilpers (1998) and performed a validity and reliability analysis of the questionnaire, utilizing only items pertaining to Shyness. The scale also used a 5-point Likert scale, ranging from “strongly disagree” (1) to “strongly agree” (5), with scores ranging from 1 to 5. There were a total of 3 questions, and higher scores indicated a higher level of shyness. Sample items included “I feel shy when there are other people around” and “I find it difficult to relax and be myself when I’m with others.”

### Reliability and validity analysis

In terms of internal consistency analysis for the pilot questionnaire in this study, Cronbach’s alpha coefficients for the Shyness Tendency scale, as well as the Loneliness scale, were 0.88 and 0.85, respectively. These coefficients were both greater than 0.70, indicating good reliability for each scale [75].

For exploratory factor analysis, the Kaiser-Meyer-Olkin (KMO) test and Bartlett’s test of sphericity were conducted to evaluate whether the scales were suitable for factor analysis [76, 77]. Following the suggestion of Pett et al. [78], items with factor loadings lower than 0.40 were removed. All scales in this study met this criterion. The Bartlett’s test results were as follows: Shyness Tendency scale (χ2 = 240.82, df = 21, *p* <.001), with factor loadings ranging from 0.62 to 0.86, all greater than 0.40; and Loneliness scale (χ2 = 431.74, *df* = 36, *p* <.001), with factor loadings ranging from 0.47 to 0.84, all greater than 0.40.

Regarding the internal consistency of the formal questionnaire in this study, Cronbach’s alpha coefficients for the Shyness Tendency scale and the Loneliness scale, were 0.83 and 0.88, respectively. These coefficients were both greater than 0.70, indicating good reliability for each scale [75]. In terms of confirmatory factor analysis, several criteria were applied to assess the model fit. First, items with factor loadings below 0.45 were deleted as they did not meet the requirement for adequate fit [79, 80]. Additionally, items with close error covariances were removed based on modification indices (MI), following the evaluation criteria proposed by Jackson et al. [81] and other scholars. For the overall model evaluation, the following fit indices were considered: for the Shyness Tendency scale, the chi-square test statistic was 20.352, with 8 degrees of freedom, and the p-value was less than 0.05, indicating a significant level. However, chi-square values can be affected by sample size. Considering other fit indices, the Normed Chi-Square (NC), Standardized Root Mean Square Residual (SRMR), Comparative Fit Index (CFI) all met the standard criteria (NC = 2.544 < 3, SRMR = 0.04 < 0.08, CFI = 0.95 > 0.90), and the Root Mean Square Error of Approximation (RMSEA) was within an acceptable range (RMSEA = 0.08 < 0.10) [79]. Overall, the model fit for this measurement was acceptable. For the Loneliness scale, the chi-square test statistic was 0.453, with 2 degrees of freedom, and the p-value was less than 0.05, indicating a significant level. However, like the Shyness Tendency scale, the chi-square value can be influenced by sample size. Considering other fit indices, the Normed Chi-Square (NC), SRMR, CFI, and RMSEA all met standard criteria (NC = 0.23 < 3, SRMR = 0.01 < 0.08, CFI = 1.0 > 0.90, RMSEA < 0.001), indicating good model fit overall.

In terms of the analysis of model internal structure fit, according to Anderson & Gerbing [82], when the average variance extracted is greater than 0.50, it indicates that latent variables have ideal convergent validity. On the other hand, when the composite reliability exceeds 0.60, it indicates consistency among latent variables [83]. In this study, “Shyness Tendency” had a composite reliability of 0.88 and an average variance extracted (AVE) of 0.72, while the “Loneliness” scale had a composite reliability of 0.83 and an AVE of 0.56. These values met the acceptable standards. In other words, the questionnaire should be able to measure individual shyness tendency and loneliness traits effectively.

### Data analysis

This study employed hierarchical regression analysis to examine the impact of different independent variables on the dependent variable. First, gender was considered as a control variable and entered into the first step of the hierarchical regression to control for the influence of individual background: gender, on the dependent variable. In the second step, the independent variable, Facebook usage time, was entered, along with separate moderator variables: age (M1) and shyness tendency (M2). In the third step, interaction terms between the independent variables and moderator variables were added: Facebook usage time × age (M1) and Facebook usage time × shyness tendency (M2).

To address potential issues of collinearity arising from high correlations between independent and moderator variables, the study followed the approach proposed by Aiken et al. (1991) by centering the variables, which helps mitigate problems related to multicollinearity. Finally, the analysis examined whether the independent and moderator variables interacted to influence the dependent variable.

## Results

### Descriptive statistics and correlation analysis

This study found that, in terms of descriptive statistics, college students have an average daily total Facebook usage time of 74.64 min with a standard deviation of 58.56 min, while older adults have an average daily total Facebook usage time of 58.56 min with a standard deviation of 101.13 min. Regarding age (M1), there is a significant positive correlation between Facebook usage time and loneliness among college students (*r* =.26, *p* <.01). In contrast, the correlation between Facebook usage time and loneliness among older adults did not reach a significant level (*r* = −.124, *p* =.18). This suggests that as college students spend more time on Facebook, their levels of loneliness tend to increase, while for older adults, the relationship between Facebook usage time and loneliness is not statistically significant.

In terms of shyness tendency (M2), there is a significant positive correlation between shyness tendency and loneliness (*r* =.220, *p* <.01), indicating that individuals with a higher level of shyness tendency tend to experience higher levels of loneliness, as shown in Table 1.

**Table 1:** Descriptive statistics and correlation analysis

		Descriptive statistics				Correlation analysis		
		Min	Max	Mean	SD	X	M1	M2
Younger adults	1. Time spent on Facebook (minutes) (X)	18	300	74.64	58.56	-		
	2. Age (M1)	18	25	19.40	1.33	0.21^*^	-	
	3. Shyness tendency (M2)	1.33	4.67	3.18	0.82	0.03	0.13	-
	4. Loneliness	1.00	4.11	1.92	0.56	0.25^**^	0.27^**^	0.14
Older adults	1. Time spent on Facebook (minutes) (X)	6	720	85.38	101.13	-		
	2. Age (M1)	50	82	60.81	5.80	0.07	-	
	3. Shyness tendency (M2)	1.00	4.00	2.44	0.70	0.00	− 0.08	-
	4. Loneliness	1.00	3.22	2.10	0.51	− 0.12	0.08	0.32^**^
Overall	1. Time spent on Facebook (minutes) (X)	6	720	80.18	83.26	-		
	2. Age (M1)	18	82	40.81	21.17	0.08	-	
	3. Shyness tendency (M2)	1.00	4.67	2.80	0.84	− 0.02	− 0.44^**^	-
	4. Loneliness	1.00	4.11	2.01	0.54	0.02	− 0.04	0.22^**^

^*^p <.05, ^**^p <.01

### Moderation effects of age

**Fig. 1:**
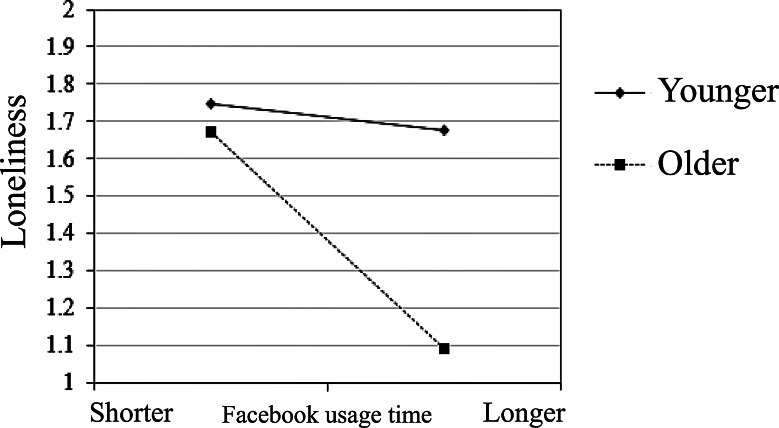
Moderating effect of Facebook usage time and age on loneliness

This section aims to test Hypothesis 1 (H1): Age moderates the relationship between individual social media usage and loneliness. As shown in Table 2, the interaction term between total Facebook usage time and age reaches a significant standard (*β*= − 0.16, *p* <.05). To further understand the interaction effects of total Facebook usage time and age on loneliness, this study divided participants into two groups, high and low, for both total Facebook usage time and age, based on the mean plus or minus one standard deviation. Subsequently, interaction plots were created to illustrate these effects, as shown in Fig. 1.

**Table 2:** The moderating effect of age on Facebook usage time and loneliness

Variables	Loneliness			
	Model A (β)	Model B (β)	Model C (β)	VIF
Step 1				
Gender	0.23^**^	0.20^**^	0.19^**^	1.00
Step 2				
Time spent on Facebook (minutes) (X)		0.39	0.10	1.04
Age (M1)		0.32^***^	0.32^***^	1.39
Step 3				
X * M1			− 0.16^*^	1.78

^*^p <.05 ^**^p <.01 ^***^p <.001

From Fig. 1, it can be observed that age moderates the relationship between individual social media usage and loneliness. Both young and older individuals experience a decrease in loneliness as their Facebook usage time increases. In the case of young individuals, the differences are not substantial, but for older individuals, loneliness significantly decreases as their usage time on Facebook increases. These findings partially support H1.

### Moderation effect of shyness

**Fig. 2:**
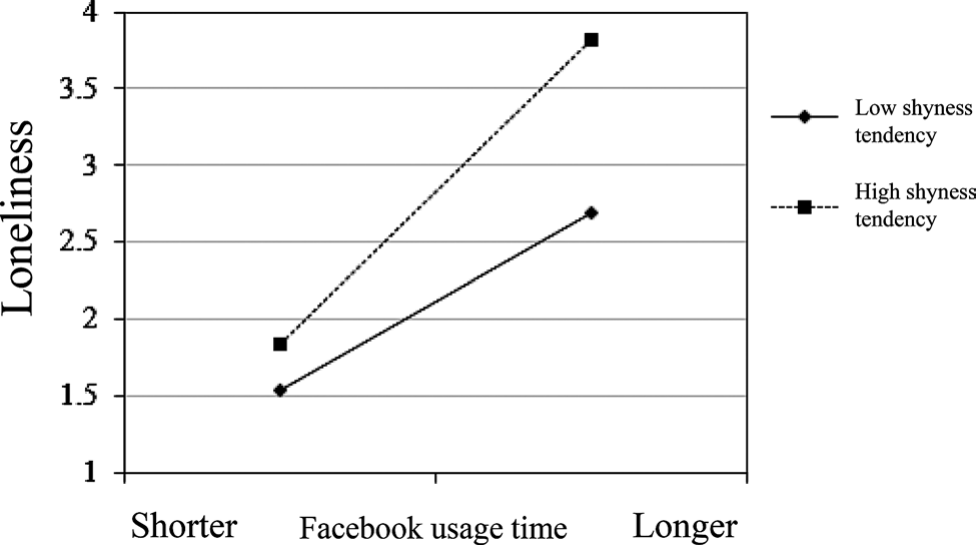
Moderating effect of Facebook usage time and shyness tendency on loneliness

This section aims to verify Hypothesis 2 (H2): Shyness tendency moderates the relationship between individual social media usage and loneliness. As shown in Table 3, the interaction term between total Facebook usage time and shyness tendency significantly positively predicts (*β* = 0.15, *p* <.05). To further understand the interaction effect of total Facebook usage time and shyness tendency on loneliness, this study dividedthe high and low groups of Facebook usage time and shyness tendency by adding or subtracting one standard deviation from the mean and presents the interaction effect graphically, as shown in Fig. 2.

**Table 3:** The moderating effect of shyness tendency on Facebook usage time and loneliness

Variables	Loneliness			
	Model A (β)	Model B (β)	Model C (β)	VIF
Step 1				
Gender	0.23**	0.20**	0.19**	1.00
Step 2				
Time spent on Facebook (minutes) (X)		0.39	0.10	1.04
Shyness tendency (M2)		0.37***	0.38***	1.30
Step 3				
X * M2			0.15*	1.49

^*^p <.05 **p <.01 ***p <.001

From Fig. 2, it can be observed that overall, shyness tendency moderates the relationship between individual social media usage and loneliness. Compared to individuals with low shyness tendency, those with high shyness tendency experience a greater increase in loneliness as their usage time on Facebook lengthens. The results of this study partially support Hypothesis H2.

## Discussion

### Moderation effect of age on social media usage and loneliness relationship

In terms of age, this study found that both young people and older adults experience a decrease in loneliness as their Facebook usage time increases. However, the difference in young people is not significant, whereas older adults experience a substantial decrease in loneliness with longer Facebook usage time.

Regarding the relationship between social media usage and loneliness in older adults, the results of this study align with past research. Heo et al. [84] studied the internet usage patterns of 65-year-old older adults and found that increased internet usage was associated with reduced loneliness, better social support, increased life satisfaction, and improved psychological well-being. Khalaila and Vitman-Schorr [85] researched internet usage among 502 individuals aged 50 and above and similarly found that internet usage reduced loneliness and directly or indirectly enhanced the quality of life for older adults.

However, when it comes to the relationship between social media usage and loneliness in young people, the results have been inconsistent, with some studies aligning with this study’s findings. Lou et al. [86] examined the relationship between Facebook usage intensity and loneliness among college freshmen. Facebook usage intensity refers to the level of emotional investment users had in Facebook, with higher intensity indicating greater emotional involvement and longer time spent on Facebook. They found that greater Facebook usage intensity was associated with reduced loneliness.

Past research has generally shown that spending more time online is associated with increased negative emotions such as depression and loneliness among young people [8, 9]. From the above, this study’s results partially support its hypothesis. The study speculates that the reason for the substantial decrease in loneliness in older adults with longer usage time might be because college students have more diverse social interactions. Apart from using social media to connect with real-life friends and online friends [87], they continue to interact with others in their daily lives [88]. Therefore, the influence of Facebook may be relatively smaller for young people, resulting in minimal differences in the relationship between Facebook usage time and loneliness. In contrast, older adults use Facebook mainly to enhance and maintain existing relationships [89]. Thus, using social media to strengthen their existing social connections may enhance social support, life satisfaction, and significantly reduce loneliness [84, 85].

### Moderating effect of shyness on the relationship between Facebook usage and loneliness

In terms of shyness, shyness moderates the relationship between individual social media usage and loneliness. Both individuals with high and low shyness experience an increase in loneliness with longer Facebook usage time, but the increase is more pronounced among individuals with high shyness.

Regarding individuals with low shyness and their Facebook usage patterns, this study’s results are consistent with past research. Bian and Leung [70] studied smartphone addiction and found that individuals who spent more time browsing social networking sites, receiving and sending messages, which implies heavy smartphone use, were more likely to be shy and experience higher levels of loneliness. Similarly, Satici [71] found that individuals addicted to Facebook, especially those with higher shyness tendency, reported reduced subjective well-being. In other words, individuals with higher shyness tendency experience an increase in loneliness with longer Facebook usage time.

However, for individuals with low shyness, this study’s results do not align with past research. Previous studies have indicated that Facebook users tend to be more extroverted and less lonely [72]. Additionally, Zhou et al. [73] studied the online behavior of introverted and extroverted individuals on social media and found that extroverted individuals were more likely to express both positive and negative emotions online. In contrast, introverted individuals tended to post more content related to negative emotions, expressing anger, fear, and disgust. In other words, individuals with low shy tendency can effectively express both positive and negative emotions, leading to a reduction in loneliness.

This study found that individuals with low shyness tendency experience an increase in loneliness with longer Facebook usage time, although the increase is relatively small. From the perspective of social comparison theory, individuals tend to engage in self-assessment by comparing themselves with others in the absence of objective comparison standards [48]. Facebook is a publicly accessible non-anonymous social media platform where users present idealized versions of themselves. Consequently, everyone may perceive others’ lives as happier, leading to a comparative mindset [90] and negative emotions [91]. Therefore, individuals with low shyness tendency may also experience an increase in loneliness with longer Facebook usage time. On the other hand, individuals with high shyness tendency may be more prone to prolonged social media addiction [70, 92], leading to a stronger sense of loneliness.

### Limitations and implications

This study contributes to academia by providing scholars with insights into the usage patterns, basic characteristics, and personality traits of social media users. This understanding helps identify the factors influencing loneliness. In terms of social media platform operation: (1) Through these analytical findings, social media platform operators can gain a deeper understanding of user profiles and personalities. This insight can help them understand how users engage with the platform. It also highlights that certain user types may experience more negative emotions when using social media. Armed with this knowledge, operators can focus on improving or adjusting the platform in these areas. (2) The results of this study reveal that shyness plays a significant role in moderating the association between Facebook usage and feelings of loneliness. Therefore, in the future, platform operators may be able to reduce negative emotions by addressing users’ comparative mindsets and fostering self-affirmation. For instance, they could consider measures such as removing the “like” button on social media. (3) Furthermore, this research indicates that age is a factor in moderating the relationship between Facebook usage and loneliness. Older users who use social media more frequently experience lower levels of loneliness. Therefore, in the future, platform operators can design more features or activities that are relevant to older users and extend online interactions into real-life situations. This approach can strengthen older users’ appreciation of social media platforms.

In terms of Facebook usage patterns, this study faced constraints related to human resources, time, and budget considerations, which prevented the use of a random sampling procedure to obtain a representative sample. Therefore, the data collected may not be fully representative. For example, the sample in this study primarily consisted of young adults who are university students, while the age range of older participants was broader. Regarding the age of the older participants, this study included individuals aged between 50 and 82 years. Smith and Anderson [93] found that even among older individuals, there are variations in Facebook usage. The usage rate for Facebook among people aged 50 to 64 is 68%, while it drops to 46% for those aged 65 and above. Furthermore, when considering the number of friends on social media, Hutto et al. (2015) noted that younger older users (aged 50–64) tend to have more friends compared to older users (aged 65–91).

Therefore, future research could explore Facebook usage patterns among older individuals in greater detail by differentiating between age groups, such as individuals aged 50–64 and those aged 65 and above in order to investigate their potential differences. In terms of motivations for Facebook usage, the questionnaires in this study were distributed to both older adults and university students. However, due to concerns about the willingness of older adults to complete extensive questionnaires, the study was not able to comprehensively investigate the reasons for using Facebook. Future research could address this limitation by delving deeper into Facebook usage patterns, such as examining the total number of Facebook friends, and by exploring the motivations for using Facebook, including seeking popularity, emotional expression, information seeking, entertainment, and time-passing activities [69], in relation to feelings of loneliness.

Last but certainly not least, the present study utilizes a cross-sectional research design, which limits our ability to observe how the causal effect between variables. Considering that both social media usage and feelings of loneliness may undergo dynamic changes over time, it is advisable for future researchers to explore longitudinal study designs or employ methods like experimental designs to capture data at various time points. This approach would enhance the depth of research findings in the relevant field.

## Conclusion

This study has two main findings. Firstly, it was discovered that among older individuals, spending more time on Facebook significantly reduces feelings of loneliness. This can potentially alleviate the issue of social isolation that often affects older adults [10, 11]. Facebook usage allows older individuals to rebuild bridges of social interaction, expanding and enriching their lives. Secondly, in terms of shyness tendency, individuals with higher levels of shyness experience a greater increase in feelings of loneliness as their time on Facebook increases, in comparison to those with lower shyness tendency [71, 94]. However, both groups may experience increased loneliness due to the potential for a comparative mindset on Facebook [90], which can lead to negative emotions [91]. Therefore, it is evident that individuals, whether they have high or low shyness tendency, should use Facebook with a positive attitude and in moderation to promote a more positive life experience, rather than fostering negative emotions.

## Data Availability

The raw data supporting the conclusions of this article will be made available by the authors, without undue reservation. The data are not publicly available due to restrictions their containing information that could compromise the privacy of research participants.
